# 多发性骨髓瘤应用二代测序监测微小残留病的实验室标准化技术规范专家共识（2021年版）

**DOI:** 10.3760/cma.j.issn.0253-2727.2021.12.002

**Published:** 2021-12

**Authors:** 中华医学会血液学分会实验诊断学组、中华医学会血液学分会浆细胞疾病学组

多发性骨髓瘤（multiple myeloma，MM）是一种细胞遗传学高度异质的克隆性浆细胞增殖性肿瘤，是血液系统的常见肿瘤[1]。近几年多种新型作用机制靶向药物的上市大大提高了MM患者治疗的缓解深度并延长了生存期。但由于大部分患者体内存在微小残留病（minimal residual disease，MRD），最终仍会复发。二代测序（next-generation sequencing，NGS）作为新的分子生物学技术，具有通量高、灵敏度高、成本低等优势，已成为MM患者MRD评估的主要研究方法之一。为实现检测结果标准化，专家组制订了在MM患者中应用NGS监测MRD实验室标准化技术规范的中国专家共识。

一、背景

1. MRD监测意义：MRD可在低于传统形态学检测多个数量级下检测恶性肿瘤是否持续存在，是评估肿瘤负荷的常用指标，可反映患者对治疗的反应，也可作为评估患者未来复发风险的预后工具。国际骨髓瘤工作组（International Myeloma Working Group，IMWG）在2016年更新了MM治疗应答标准，在原有传统疗效评估标准之外增加了MRD疗效标准，主要包括二代流式细胞术和NGS两种监测方法[2]–[3]。2020年版中国MM诊治指南也推荐将NGS纳入MM患者的MRD评估标准。国内外文献也证实NGS检测的MRD状态可作为MM的主要预后因素之一，是MM患者疗效评价的重要标准[4]–[6]。

2. MRD目标基因：目前应用NGS监测MM患者MRD，主要检测的目标基因是IG基因克隆性重排。需要先确定初诊MM患者全骨髓细胞中肿瘤浆细胞IG克隆性重排，包括IGH和（或）IGK基因克隆性重排的类型和序列[7]。在患者后续的随访标本中，以诊断时检测到的克隆性重排序列作为分子标志进行MRD监测[8]。

二、实验流程

1. 样本采集：MM患者建议采集新鲜骨髓标本进行NGS检测，不推荐使用外周血。如因某些原因不能采集到新鲜标本，也可使用冻存标本，建议在−80 °C及以下保存并尽快送检，防止DNA降解。对于初诊患者，骨髓采集量以1～3 ml为宜。对于缓解患者，骨髓采集量以3～5 ml为宜，若白细胞计数偏低，应适当调整采集量，使有核细胞总数达到1×10^7^个以上。骨髓标本采集管要求EDTA或枸橼酸钠抗凝，禁止使用肝素抗凝。如外地运输标本，推荐72 h内4 °C冷藏运送并具备相应物流质控体系[9]。

2. 核酸提取：DNA质量是NGS检测是否成功的关键因素。样本处理可采用红细胞裂解液离心沉淀全部有核细胞。提取有核细胞中的核酸DNA，DNA提取方法可以使用过滤柱法、磁珠法等，推荐使用商品化的DNA抽提试剂盒。抽提完成的核酸必须评估其质量，评价指标包括DNA浓度、纯度和完整性。RNA、蛋白、无机盐、各种杂质的污染及DNA的降解程度均会影响最终检测结果的灵敏度。因此，实验室应设立明确的合格DNA样本的判断标准[9]。DNA完整性评估可使用琼脂糖凝胶电泳、安捷伦基因组 DNA ScreenTape分析等。

3. 文库制备：文库制备用于检测是否目标区域的富集，常用的方法有杂交捕获法和扩增子法。针对IG重排的特殊性，目前国内外普遍采用扩增子法建库，即设计一系列引物，多重PCR扩增目的区域片段。对于多样本同时检测可以加入不同的分子标签以区分多个不同的检测标本。可以使用商品化的NGS重排检测试剂盒[10]。

4. 文库定量：文库质量可直接影响最终测序数据的质量。如文库浓度过高可导致多克隆数据的产生，降低有效测序数据量；过低则可导致整体测序数据的减少，因此，在测序之前需要进行文库的定量。目前常用的方法有Qubit荧光计、实时荧光定量PCR等。推荐使用实时荧光定量PCR方法，可使用NGS定量PCR检测试剂盒，如针对Ion Torrent平台可使用Applied Biosystems公司的Ion Library® TaqMan Quantitation Kit，针对illumina平台可使用KAPA公司的KAPA Library Quantification Kit。

5. 上机测序：目前国内实验室通常采用的NGS测序平台有三大类：Illumina、Thermo Fisher、MGI，每个平台有不同型号的配置设备。测序检测前需根据测序平台确定相应的数据参数，并根据测序片段的长度、检测标本的数量、标本的质量和最低测序深度等因素选用合适的芯片，以保证本次测序结果数据质量合格，能够分析出最低检测下限阳性对照标本[11]。

三、生物信息学分析流程：

1. 质控分析：对下机数据进行质量评估，制定有效的质量控制标准，包括测序数据质量、测序覆盖度、阴性和阳性标本检测结果等评价参数。如S5或PGM测序平台质量标准包括装载量>50％、富集率>50％、克隆数>50％；Miseq测序平台质量标准包括V2试剂PE250测序碱基的质量Q30>75％、V3试剂PE300测序碱基的质量Q30>70％。

2. 数据过滤：需要明确对测序数据的过滤标准，包括对测序质量低的序列、长度不完整的序列、每张芯片由于接头污染引起标本之间交叉污染等进行过滤[12]。针对IG重排需要过滤掉缺少V区或J区的序列、无法组装成完整克隆类型的序列。

3. 序列比对：IG重排比对不同于常规NGS使用的人类基因组参考序列，需要使用专门的基因重排参考序列库。推荐使用NCBI、IMGT数据库或实验室自建数据库对下机序列进行比对，也可以使用商品化试剂配套的一站式分析流程软件[13]。

四、质量控制

1. 常规要求：实验中需建立完整的SOP文件，包括标本采集和处理、实验操作、生物信息学分析和报告分析等各个环节的SOP文件，实验操作程序和生物信息学分析须经过性能验证或确认。其他未说明的参照NGS检测技术实验室质量控制与管理的常规要求[9]。

2. MM患者MRD检测的特殊要求：

（1）设立对照样本：同批次标本检测需同时加入相应的质控标本，如空白对照、阴性对照、最低检测下限阳性对照和高比例阳性对照。针对MM患者的MRD检测，需要至少设立4种质控样本：①空白对照；②阴性对照；③弱阳性对照，如10^−4^检测限的前批次标本；④内参质控[14]。

（2）样本总量：对于初诊MM患者，推荐合格DNA样本的总量至少达到250 ng，以确保能够检测到肿瘤性浆细胞的克隆性重排。对于缓解期的MM患者，推荐NGS检测MRD的合格DNA上样总量至少达到3 000 ng[15]。

（3）测序数据量：制定每个检测样本测序数据量是否合格的判断标准。例如，对于初诊标本，LymphoTrack方法检测最终得到的有效读段（reads）数需大于10 kb，小于10 kb为实验失败，需重新测序或重建文库。对于MRD检测标本，总reads数至少达到所设定的MRD检测敏感度的下限。

（4）性能确认：不同实验室应提供各自建立的MRD检测方法的实验室验证和标准化信息，对于实验操作的各个环节均需建立相应的SOP文件，实验方法须经过性能验证或确认[16]。至少包括准确度、灵敏度、可重复性、最低检测限和可报告范围，以及相应数据的计算方法。NGS检测MRD实验方法的灵敏度主要由样本投入量和测序数据量共同决定。可根据所建立的实验方法，利用统计学分析，计算该MRD检测技术体系的置信度。图1为基于LymphoTrack实验方法、统计学计算得出95％置信度下MRD阴性的模型。由此得出在不同灵敏度下MRD阴性所推荐的数据标准（表1）。国内外研究指南推荐，MM患者的MRD检测灵敏度至少达到10^−5^，即10^5^个有核细胞中可检测出1个克隆性浆细胞[3],[5]–[6]。

**图1:**
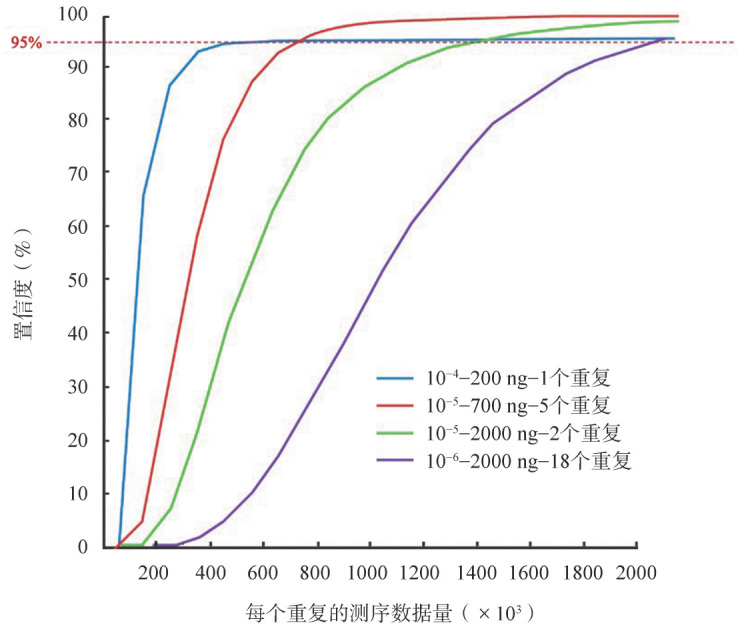
微小残留病阴性的置信度预测模型

**表1:** 不同灵敏度下微小残留病阴性推荐的数据标准

灵敏度	DNA输入量（ng）	总测序数据量（reads)
1×10^−4^	200	500 000
1×10^−5^	2 000	4 400 000
1×10^−6^	20 000	44 400 000

3. 室间比对：实验室应定期参加MRD检测项目的室间质量评价或能力验证；如因该MRD检测项目目前尚无参比方法，可通过与其他使用相同检测系统的实验室进行比对的方式判断该MRD检验结果的可接受性。推荐初诊和缓解标本在同一所实验室检测，以保证检测结果的可比性和一致性。

五、报告单内容

对于MRD监测报告单，推荐结合初诊标本检测结果和MRD监测结果进行比较并作相应解释说明。在报告MRD数据时，应说明每个样品的检出限。对于MRD阴性，建议使用术语uMRD（未检测到MRD）；对于MRD阳性，建议提供可检测MRD的定量结果[17]。

推荐MRD报告单内容至少应包括：①DNA上样总量；②目标基因；③MRD监测的克隆性重排类型、重排比例；④本次检测方法的灵敏度；⑤对结果进行解释说明；⑥有条件的实验室可列出每次MRD监测的时间点，并绘制相应MRD结果的动态变化曲线。肿瘤细胞克隆性重排序列比例计算方式为：肿瘤细胞克隆性重排序列总数/（肿瘤细胞克隆性重排序列总数+正常细胞重排序列总数）[14]。

其他涉及二代测序实验技术的基本要求和规范，包括LDT自建项目要求、生物信息学分析、质量控制和管理、报告单内容等推荐参考相关行业的推荐和共识[18]–[20]。
